# Molecular–genetic and clinical characteristics of gliomas with astrocytic appearance and total 1p19q loss in a single institutional consecutive cohort

**DOI:** 10.18632/oncotarget.3869

**Published:** 2015-05-11

**Authors:** Saeko Hayashi, Hikaru Sasaki, Tokuhiro Kimura, Takayuki Abe, Takumi Nakamura, Yohei Kitamura, Tomoru Miwa, Kaori Kameyama, Yuichi Hirose, Kazunari Yoshida

**Affiliations:** ^1^ Department of Neurosurgery, Keio University School of Medicine, Shinanomachi, Shinjuku-ku, Tokyo, Japan; ^2^ Department of Pathology, Keio University School of Medicine, Shinanomachi, Shinjuku-ku, Tokyo, Japan; ^3^ Center for Clinical Research, Department of Preventive Medicine and Public Health, Keio University School of Medicine, Shinanomachi, Shinjuku-ku, Tokyo, Japan; ^4^ Department of Neurosurgery, Saiseikai Utsunomiya Hospital, Takebayashi, Utsunomiya, Tochigi, Japan; ^5^ Division of Diagnostic Pathology, Keio University School of Medicine, Shinanomachi, Shinjuku-ku, Tokyo, Japan; ^6^ Department of Neurosurgery, Fujita Health University School of Medicine, Kutsukake-cho, Toyoake, Aichi, Japan; ^7^ Present address: Department of Pathology, Yamaguchi University Graduate, School of Medicine, Minami-kogushi, Ube, Yamaguchi, Japan

**Keywords:** total 1p19q loss, 1p19q codeletion, astrocytic, ATRX, p53

## Abstract

The prognostic significance of 1p19q loss in astrocytic gliomas has been inconclusive.

We collected 57 gliomas with total 1p19q loss from among 218 cases of WHO grade-II/III gliomas operated at Keio University Hospital between 1990 and 2010. These tumors were classified as oligodendroglial or “astrocytic” by a WHO-criteria-based institutional diagnosis. Chromosomal copy number aberrations (CNAs), *IDH 1/2* mutations, *MGMT* promoter methylation, and expression of p53 and ATRX were assessed. Survival outcome was compared between the two histological groups.

Of the 57 codeleted gliomas, 37, 16, and four were classified as oligodendroglial, “astrocytic”, and unclassified, respectively. Comparative genomic hybridization revealed that although chromosome 7q/7 gain was more frequent in “astrocytic” gliomas, other CNAs occurred at a similar frequency in both groups. None of the “astrocytic” gliomas showed p53 accumulation, and ATRX loss was found in three of the 15 “astrocytic” gliomas. The estimated overall survival (OS) curves in the patients with codeleted oligodendroglial and “astrocytic” gliomas overlapped, and the median OS was 187 and 184 months, respectively. Histopathological re-assessment by a single pathologist showed consistent results.

Gliomas with total 1p19q loss with “astrocytic” features have molecular and biological characteristics comparable to those of oligodendroglial tumors.

## INTRODUCTION

Since the initial discovery of the predictive and prognostic significance of chromosome 1p loss [1], evidence has accumulated suggesting that the total loss of chromosomes 1p and 19q (resulting from an unbalanced centromeric translocation t (1;19) (q10; p10)), is associated with increased chemosensitivity and longer survival in oligodendroglial gliomas [2-9]. Although total 1p19q loss is a genetic hallmark of oligodendroglial tumors, it is also occasionally observed in astrocytic gliomas [10-16]. Most previous studies that have demonstrated a predictive and prognostic relevance of 1p19q loss have involved anaplastic oligodendroglial tumors. In contrast, the publication of similar studies involving 1p19q codeleted astrocytic gliomas is limited, and the conclusions reached have been inconclusive [16-18]. Furthermore, the morphological distinctions between non-classic oligodendroglioma and astrocytoma suffers from significant interobserver variation and is thus associated with limited reproducibility [4, 19]. Therefore, whether the clinical and biological significance of 1p19q loss can be generally applied to diffuse gliomas beyond histological classification remains to be validated.

In this study, we investigated a consecutive series of diffuse gliomas with an “astrocytic” appearance and that exhibited 1p19q loss, in order to assess their molecular–genetic and clinical–biological characteristics. The deletion status of 1p and 19q was analyzed by comparative genomic hybridization (CGH), which (unlike fluorescence in situ hybridization (FISH)) can accurately distinguish between complete and partial chromosome loss [16, 20]. The molecular–genetic and clinical–biological characteristics of “astrocytic” gliomas with total 1p19q loss were compared with those of oligodendroglial gliomas on the basis of the original institutional diagnosis as well as diagnosis by a single pathologist. This was done to exclude bias due to inter-observer variability or due to changes in diagnostic criteria over time [19, 21]. Moreover, because the study population was collected from consecutive cases treated at a single institution over two decades, we were able to determine the Japanese incidence of diffuse gliomas with 1p19q loss.

## RESULTS

### Histopathological distribution of gliomas with total 1p19q loss

In total, 218 supratentorial, diffuse gliomas of WHO grade II or III from adult patients without prior chemotherapy or radiotherapy were found in the hospital pathology records. The status of 1p and 19q was examined for clinical purpose in 152 of 218 gliomas. Of these, 53 were oligodendroglial (oligodendroglioma, oligoastrocytoma, anaplastic oligodendroglioma, or anaplastic oligoastrocytoma), 93 were astrocytic (astrocytoma or anaplastic astrocytoma), and 6 were unclassified because of small sample size, by the original institutional diagnosis. Fifty-eight of 152 gliomas (38.2%) showed 1p19q loss. Of these, 38 were oligodendroglial (71.7% of all oligodendroglial tumors), 16 were astrocytic (17.2% of all astrocytic tumors), and 4 were unclassified. Written informed consent for translational research (institutional approval number 20050002) was obtained for 57 of the 58 codeleted gliomas, and subsequent analysis was performed on these 57 tumors. The 57 patients comprised 35 males and 22 females, with ages ranging from 22 to 63 years. Demographic factors such as age and sex were comparable between the oligodendroglial and astrocytic groups (Table S1).

The 37 codeleted oligodendroglial gliomas comprised 15 oligodendrogliomas, 9 oligoastrocytomas, 9 anaplastic oligodendogliomas, and 4 anaplastic oligoastrocytomas (Table S1). Nine of the 37 cases were diagnosed by biopsy.

The 16 codeleted “astrocytomas” comprised 7 diffuse astrocytomas and 9 anaplastic astrocytomas. Five of the 16 cases were diagnosed by biopsy (Table S1).

Histopathological features of the 57 codeleted gliomas were re-assessed by a single pathologist according to current criteria [22]. Following re-assessment, 40 were classified as oligodendroglial and 15 as “astrocytic,” while 2 were unclassified because of small sample size.

### Molecular–genetic results

Of the 57 cases, CGH profiles were obtained for all, *IDH* mutation status was obtained for 53, and *MGMT* promoter methylation status was obtained for 51. Evaluation of the status of ATRX and p53 were possible in 15 of the 16 codeleted “astrocytic” gliomas. Mutational screening results along with original institutional diagnoses for each case are found in Table S1.

**Figure 1:**
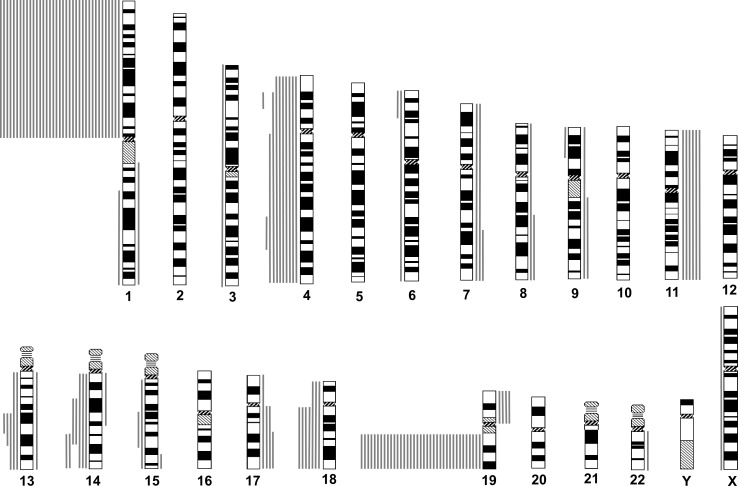
Chromosomal copy number aberrations (CNAs) of oligodendroglial gliomas with total 1p19q loss, as determined by institutional diagnosis Lines to the left of each idiogram represent regions of reduced relative DNA copy number, and lines to the right represent regions of increased relative DNA copy number. Each line represents a CNA found in one tumor.

Among the 37 oligodendroglial gliomas, 26 revealed CNAs besides the 1p19q codeletion. The most frequent was loss on chromosome arm 4q (9 cases, 24.3%), followed by loss on 14q (7 cases, 18.9%), loss of 18q (7 cases, 18.9%), gain on 11q/11 (gain of 11q or total gain of 11, 6 cases, 16.2%), loss on 13q (5 cases, 13.5%), gain on 17q (4 cases, 10.8%), and gain on 19p (4 cases, 10.8%) (Figure 1, Table 1). *IDH1* or *IDH2* mutations were detected in all 34 cases (100%) for which both genes were successfully examined. All changes at *IDH1* codon 132 comprised a CGT to CAT (R132H) mutation, and all *IDH2* codon 172 mutations were AGG to ATG (R172M). Twenty-one of 32 analyzed cases (65.6%) displayed *MGMT* promoter methylation (Table 1).

**Table 1:** Summary of chromosomal copy number aberrations (CNAs) and status of *IDH* and *MGMT* genes in gliomas with total 1p19q loss by institutional diagnosis

	Oligo (n=37)	Astro (n=16)	Unclassified low-grade glioma (n=4)
CNAs (detected in ≥10%)	−1p19q (100%) −4q (24.3%) −14q (18.9%) −18q (18.9%) +11q or +11 (16.2%) −13q (13.5%) +17q (10.8%) +19p (10.8%)	−1p19q (100%) +7q or +7 (31.2%) −4q (25%) +11q or +11 (18.8%) −18q (18.8%) +19p (18.8%) −9p (12.5%) +9q (12.5%) −10p (12.5%) −14q (12.5%) −15q (12.5%) +17q (12.5%)	−1p19q(100%) −4(25%) −19p(25%)
IDH 1 mutation	31/36(86.1%)	12/13(92.3%)	4/4(100%)
IDH 2 mutation	3/3(100%) (Either IDH1 or IDH2 mutations in 34/34, 100%)	0/1(0%) (Either IDH1 or IDH2 mutations in 12 of 13, 92.3%)	Not performed
MGMT methylation	21/32(65.6%)	15/15(100%)	2/4(50%)
ATRX loss	Not performed	3/15(80%)	Not performed
P53 accumulation	Not performed	0/15%(0%)	Not performed

**Figure 2:**
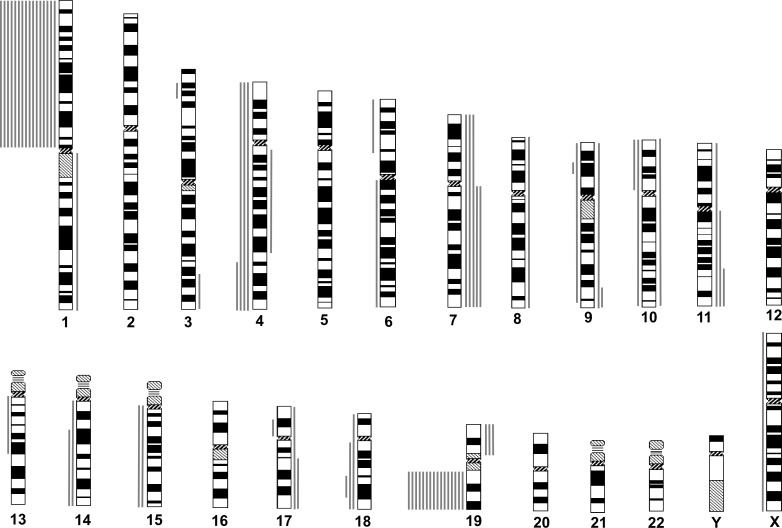
Chromosomal copy number aberrations (CNAs) of astrocytic gliomas with total 1p19q loss, as determined by institutional diagnosis Idiogram features are as described for Figure. 1.

Among the 16 “astrocytic” gliomas, 12 revealed CNAs besides the 1p19q codeletion. The most frequent were gain on chromosome 7q/7 (gain on 7q or total gain of 7, 5 cases, 31.2%), loss on 4q (4 cases, 25%), gain on 11q/11(3 cases, 18.8%), loss on 18q (3 cases, 18.8%), gain of 19p (3 cases, 18.8%), loss on 9p (2 cases, 12.5%), gain on 9q (2 cases, 12.5%), loss of 10p (2 cases, 12.5%), loss on 14q (2 cases, 12.5%), loss of 15q (2 cases, 12.5%), and gain on 17q (2 cases, 12.5%) (Figure 2, Table 1). Mutations in *IDH1* or *IDH2* were detected in 12 of 13 cases (92.3%) for which both genes were successfully examined. *MGMT* promoter methylation was found in all 15 cases for which MSP was successfully performed. Expression of p53 was negative in all of the 15 successfully evaluated cases, whereas loss of ATRX expression was detected in three of the 15 (Table 1).

Among the four gliomas that were unclassified because of small sample size, two cases had CNAs besides the 1p19q codeletion. These comprised loss on 4 (25%) and loss of 19p (25%).

Gain on 7q/7 was significantly more frequent in “astrocytic” gliomas (*p* = 0.0015), and loss of 10p was numerically more frequent in “astrocytic” gliomas (*p* = 0.0871).

### Survival analysis

**Figure 3:**
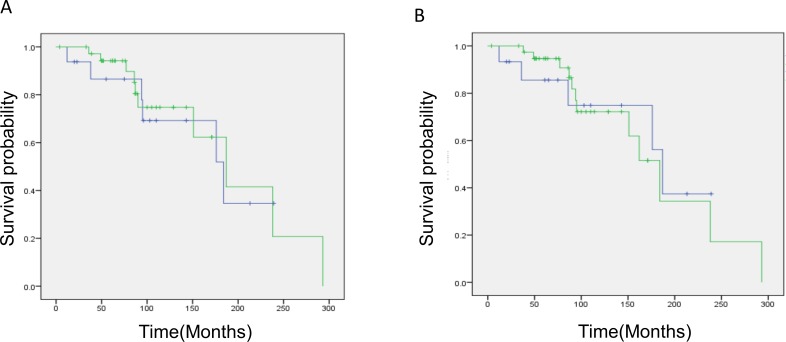
Kaplan–Meier survival analysis of oligodendroglial and astrocytic gliomas with total 1p19q loss **a.** by institutional diagnosis: The green line represents oligodendroglial tumors and the blue line represents astrocytic tumors. Median OS: oligodendroglial 187 months, astrocytic 184 months (*p* = 0.828). **b.** by single pathologist diagnosis: The green line represents oligodendroglial tumors and the blue line represents astrocytic tumors. Median OS: oligodendroglial 184 months, astrocytic 187 months (*p* = 0.84). Note that there is no difference in survival estimation between the two histological groups.

To determine whether any distinct clinical or biological characteristics occurred in 1p19q codeleted gliomas with astrocytic features, the survival outcome was compared between the two groups. Although initial treatments varied (resection alone: 11 cases in oligodendroglial versus 1 case in astrocytic; radiotherapy alone: 6 in oligodendroglial versus 5 in astrocytic; chemotherapy alone: 11 in oligodendroglial versus 1 in astrocytic; chemoradiotherapy: 9 in oligodendroglial and 9 in astrocytic), treatment intensities during the overall clinical courses were similar between the two histological groups, i.e., 22 of 37 patients with oligodendroglial gliomas and 12 of 16 patients with “astrocytic” gliomas underwent both chemotherapy and radiotherapy in their clinical courses. Kaplan–Meier survival curves overlapped between the groups, and with a median follow-up of 87 months, the median OS in the oligodendroglial and “astrocytic” glioma groups was 187 and 184 months, respectively (Figure 3a, *p* = 0.828). Eleven patients with “astrocytic” gliomas by resection showed comparable survival times (median OS: 176 months, *p* = 0.5905). Moreover, histological grouping by a single pathologist using current criteria demonstrated a consistent result, again showing no significant OS difference between the two glioma categories (Figure 3b; *p* = 0.84). Furthermore, those results were confirmed by a comparison between oligodendroglial (33) and “astrocytic” (10) gliomas by consensus of institutional and single-pathologist diagnoses (*p* = 0.67).

### Illustrative cases of “astrocytic” gliomas with total 1p19q loss

**Figure 4:**
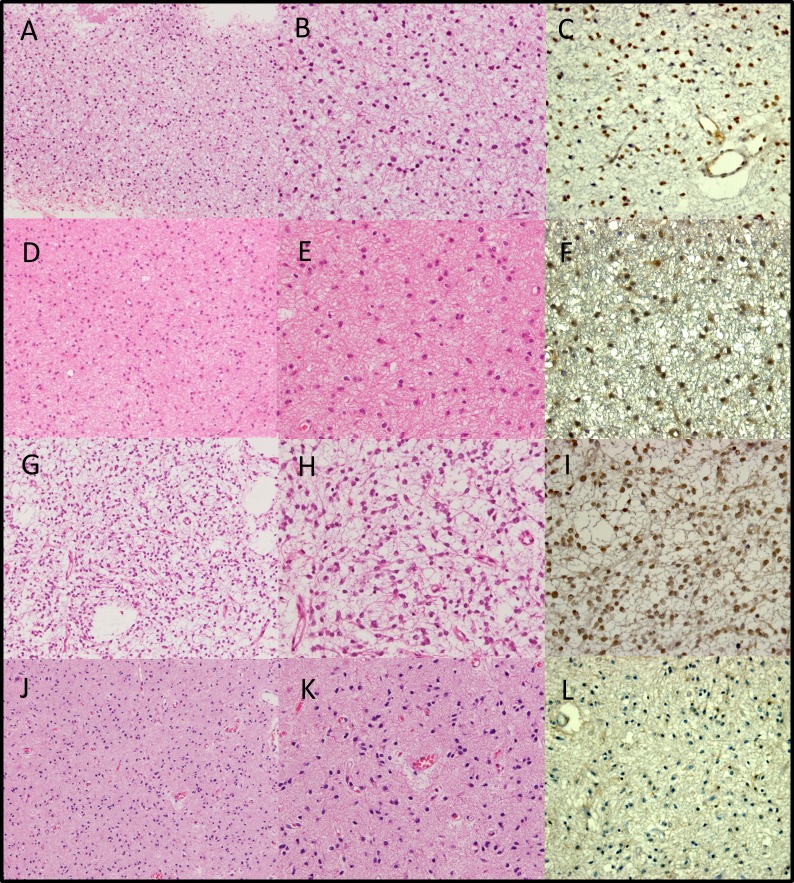
Hematoxylin and eosin staining (a, b, d, e, g, h, j, k) and ATRX staining (c, f, i, l) of examples of “astrocytic” gliomas with total 1p19q loss **a, b, c.** Table S1, case 39. Institutional diagnosis: diffuse astrocytoma. Single-pathologist diagnosis: diffuse astrocytoma. **d, e, f.** Table S1, case 44. Institutional diagnosis: diffuse astrocytoma. Single-pathologist diagnosis: diffuse astrocytoma. **g, h, i.** Table S1, case 52. Institutional diagnosis: anaplastic astrocytoma. Single-pathologist diagnosis: diffuse astrocytoma. **j, k, l.** Table S1, case 53. Institutional diagnosis: anaplastic astrocytoma. Single-pathologist diagnosis: diffuse astrocytoma. a, d, g, j: original magnification ×100. b, c, e, f, h, i, k, l: original magnification ×200.

Case 1 (Table S1, case 39): A 37-year-old (yo) female had subtotal removal of a right frontal tumor. The institutional and single-pathologist diagnoses were both diffuse astrocytoma (Figure 4a, 4b). The nuclei of the tumor cells were round to oval in shape and irregular in size. Many tumor cells exhibited cellular processes and no mitosis was observed. The patient underwent radiation therapy of 58Gy after tumor removal. CGH revealed isolated 1p19q codeletion. An *IDH1* mutation was detected and methylation of the *MGMT* promoter was observed. Expression of ATRX was retained (Figure 4c), and expression of p53 was negative. The patient remains alive 75 months from the initial surgery.

Case 2 (Table S1, case 44): A 28 yo female underwent total removal of a left frontal tumor. The institutional and single-pathologist diagnoses were both diffuse astrocytoma (Figure 4d, 4e). The nuclei of the tumor cells were round to oval in shape and irregular in size. Cytoplasmic cellular processes were present in most tumor cells. There was no mitosis observed. CNAs revealed by CGH were −1p19q, −3p24-22, and +7q. The *MGMT* promoter was methylated. Expression of ATRX was retained (Figure 4f), and expression of p53 was negative. The patient underwent radiation therapy of 50 Gy after tumor removal. Tumor recurrence occurred 196 months after initial surgery and the pathological finding at recurrence was anaplastic astrocytoma. The patient remains alive 239 months from the initial surgery.

Case 3 (Table S1, case 52): A 27 yo female underwent partial removal of a right frontal tumor. Institutional histopathological diagnosis was anaplastic astrocytoma, while diagnosis by a single pathologist was diffuse astrocytoma (Figure 4g, 4h). The nuclei of the tumor cells were mostly oval shaped and irregular in size. The cells displayed remarkable cellular processes and no mitosis was detected. The patient underwent chemoradiotherapy (50 Gy, ranimustine = MCNU) after initial surgery. CGH revealed isolated 1p19q codeletion. Mutations in *IDH 1* and *IDH2* were not detected but the *MGMT* promoter was methylated. Expression of ATRX was retained (Figure 4i), and expression of p53 was negative. The patient was lost during follow-up because of a change in residential address. No recurrence had occurred 23 months from the initial surgery.

Case 4 (Table S1, case 53): A 28 yo male underwent subtotal removal of a left frontal tumor. Institutional histopathological diagnosis was anaplastic astrocytoma, while the diagnosis by a single pathologist was diffuse astrocytoma (Figure 4j, 4k). The nuclei of the tumor cells were oval shaped and irregular in size. Most tumor cells displayed cytoplasmic cellular processes, and mitoses were very rare. A small area with higher cellularity was detected (not shown). The patient underwent chemoradiotherapy (60Gy, ranimustine = MCNU and vincristine = VCR) after the initial surgery. CGH revealed −1p19q, +10, and −18. Mutation of *IDH1* and *MGMT* promoter methylation were detected. Expression of ATRX was lost (Figure 4l), whereas p53 accumulation was not detected. Tumor recurrence occurred 52 months after the initial surgery. At present, the patient's OS is greater than 110 months.

## DISCUSSION

Some studies that have included all glioma subtypes have suggested a positive association between the presence of 1p19q codeletion and a favorable clinical outcome not only in oligodendroglial but also among diffuse gliomas [16, 23, 24]. Indeed, a recent comprehensive analysis of 293 lower grade gliomas (LGG) by The Cancer Genome Atlas (TCGA) study group suggested that LGGs could be classified into three clinically relevant groups based on IDH and 1p19q status [25]. However, probably due to their infrequency, reports that have directly investigated the association between 1p19q codeletion and patient prognosis in astrocytic gliomas are limited, and the currently available results are inconclusive [16-18]. For example, a previous study concluded that losses on 1p and 19q marked a genetic subset of tumors diagnosed as pure grade II astrocytomas. However, whether these chromosome losses were prognostic for patients with astrocytomas was a matter of further investigation [12]. Although the number of 1p19q codeleted “astrocytic” gliomas analyzed in our study is still small, to our knowledge it nonetheless represents the largest study of such tumors. An additional advantage of our study is that 1p19q codeletion was judged by CGH. CGH is one of the most common techniques to evaluate 1p19q status [26] [27], and is superior to FISH because it can accurately distinguish between true total loss of 1p and 19q and partial loss of 1p. This is of high importance because partial loss of 1p is associated with the opposite clinical outcome [16, 20]. Although copy-neutral loss of heterozygosity can not be detected by CGH, such a situation is not the case with a total loss of 1p and 19q.

The number of oligodendroglial gliomas (as assessed by our institutional diagnosis) exhibiting total 1p19q loss (71.7%) was similar to that observed in previous studies [1, 17] and even higher than that found in phase III anaplastic oligodendroglial tumor studies [2, 9]. The number of “astrocytic” gliomas, as assessed by our institutional diagnosis (17.2%), was within the range previously reported in the literature (7.2% - 44%, average 20.8%) [10-16]. However, it might be slightly higher than expected because of a recent trend that favors oligodendroglial diagnosis [21]. Moreover, diagnoses of “astrocytic” in biopsy specimens could reflect focal heterogeneity within a single tumor [19, 28]. Therefore, the “astrocytic” gliomas, as assessed by our institutional diagnosis, may comprise a mixed population of purely astrocytic gliomas and gliomas previously designated “non-classic oligodendrogliomas” [4, 29].

### Prognosis

Our study demonstrated that the survival curves of 1p19q codeleted “astrocytic” gliomas and oligodendrogliomas overlapped and that there was no statistical difference in the median survival time between the two groups. The observed median OS of the “astrocytic” gliomas with 1p19q loss (15.5 years) is much longer than that reported for low-grade astrocytomas (5–7 years) [30, 31]. The survival outcomes of the patients with “astrocytic” gliomas by resection were similar (14.7 years). Moreover, the fact that over half of the codeleted “astrocytic” gliomas in the present study were grade III tumors suggests a favorable prognosis compared with astrocytic gliomas in general (Table S1). The frequency of *IDH* mutations and *MGMT* promoter methylation was similar in both the “astrocytic” and oligodendroglioma groups. Because these genetic/epigenetic alterations are strong prognostic factors in diffuse gliomas, their similar frequency supports the hypothesis that both tumor types are likely to exhibit similar biological characteristics.

A previous study suggested the prognostic relevance of classic oligodendroglial morphology, regardless of the 1p19q status [4]. In contrast, in our consecutive cohort, we found that the survival curves of “astrocytic” and oligodendroglial gliomas with total 1p19q loss were nearly identical. Furthermore, our results were confirmed by comparison between oligodendroglial and “astrocytic” gliomas by consensus of institutional and single-pathologist diagnosis. The reasons for this discrepancy are unclear, although we can suggest some possible reasons. First, the present study included grade II and III tumors, in contrast to the previous study that included only grade III tumors, which frequently possess more complex genomic abnormalities. Second, the possible false inclusion of tumors with partial 1p and/or 19q loss (due to inaccurate FISH analysis) in non-classic tumors could have biased the previous results toward a poorer survival outcome.

### Molecular-genetic characteristics of “astrocytic” gliomas with total 1p19q loss

Along with 1p19q codeletion, loss of chromosome 4q, loss on 14q, and loss on 18q are commonly observed chromosomal alterations in oligodendroglial tumors [32, 33]. In this study, these alterations occurred at a similar frequency in gliomas with 1p19q loss, regardless of their histological phenotype.

On the other hand, the finding that gain on chromosome 7q/7 was associated with an “astrocytic” phenotype even within codeleted gliomas was intriguing. Gain on chromosome 7q/7 is the most frequent chromosomal alteration in astrocytic tumors, as determined by CGH [10, 12, 34, 35]. Therefore, an increased frequency of 7q/7 gains could suggest that several codeleted “astrocytic” gliomas in our cohort indeed had molecular marker of astrocytic differentiation [34]. Although gain of chromosome 7 is known to be associated with the progression of gliomas [36], the survival outcomes of the patients with these tumors were comparable to those without these tumors (unpublished data).

Mutations in the *TP53* and *ATRX* genes represent genetic hallmarks of astrocytomas [37, 38]. Loss of ATRX expression in three of the 15 codeleted “astrocytic” gliomas could suggest the existence of their astrocytic nature. However, the absence or a low frequency of *TP53* and *ATRX* mutations compared with those of astrocytomas in general [37, 38] suggests that codeleted “astrocytic” gliomas in the present study were genetically similar to the codeleted oligodendrogliomas.

### Limitations of our study

The histopathological assessments conducted in this study were not performed by a panel of neuropathologists. Moreover, five of the 16 “astrocytic” gliomas were diagnosed by biopsy specimens. Therefore, it is possible that the “astrocytic” gliomas analyzed here could contain a population of “non-classic oligodendrogliomas” with some astrocytic features [4].

The present study was retrospective and treatments were not uniform. Nonetheless, the cohort analyzed here was collected from consecutive cases treated at a single institution, and the survival analyses were based on long-term follow-ups. Moreover, the comparable survival outcomes between the two groups were confirmed in “astrocytic” gliomas by resection, and by comparison of the two groups with consensus diagnoses.

In conclusion, gliomas with total 1p19q loss with some or more “astrocytic” features are likely to have comparable biological and prognostic characteristics to those with oligodendroglial features. Although some of the codeleted “astrocytic” gliomas showed molecular markers associated with astrocytic differentiation, such as 7q/7 gain and ATRX loss, the molecular and genetic characteristics of those gliomas were, overall, very similar to codeleted oligodendrogliomas. Thus, total loss of 1p and 19q is a robust molecular and prognostic marker for gliomas, regardless of their histological features. These findings further substantiate the importance of molecular classification of gliomas.

## MATERIALS AND METHODS

### Study population and tissue samples

Pathology records of brain tumors treated at the Department of Neurosurgery, Keio University Hospital between 1990 and 2010 were reviewed, and patients who fulfilled the following criteria were included: a) age > 18 years; b) supra-tentorial tumor location; c) institutional histopathological diagnosis of diffuse glioma of WHO classification grade II or III; d) no prior chemotherapy or radiotherapy treatment. The institutional histopathological diagnosis was conducted according to WHO criteria [22, 39-41].

### Informed consent

Written informed consent was obtained from all 57 patients finally included in the study for translational research approved by the Institutional Review Board at Keio University (Approval Number 20050002).

### Clinical data

Clinical data were obtained from the patients' records and included age at diagnosis, sex, extent of surgery, composition of initial postoperative therapy, time to progression, treatment at recurrence, and date of death or last contact. The survival outcome of patients lost during follow-up was updated for the present study.

### Reassessment of original histopathological diagnoses of gliomas with total 1p19q loss

The histological criteria for defining oligodendroglial tumors have changed over time, partly because of the relevance of the diagnosis to therapeutic decision-making and the estimation of prognosis [4, 21]. Although institutional diagnoses in our hospital over the past two decades have always been based on WHO criteria [22, 39-41], we were concerned that they may have been influenced by changes in diagnostic trends over time and/or by interobserver bias. Therefore, all cases with 1p19q codeletion were re-assessed by a single pathologist (TK), who was blinded to the 1p19q status, using hematoxylin and eosin (HE) staining based on the most recent WHO criteria [22]. Attention was paid to classical oligodendroglioma features such as uniform and rounded nuclei (often with small nucleoli) surrounded by perinuclear halos, and an even tissue distribution. Attention was also paid to features commonly found in astrocytic tumors, such as the oval to elongate, mildly pleomorphic, and more hyperchromatic nuclei, often with tapering eosinophilic cell processes, and irregular distribution [4, 29].

### Molecular-genetic analyses

Tumor DNA was extracted from microdissected pieces of formalin-fixed paraffin-embedded (FFPE) tissue [42]. For tissue microdissection, care was taken to exclude intermixed non-neoplastic glial/vascular cells as well as hemorrhagic/necrotic regions. This was based on HE staining and MIB-1 immunohistochemistry (Dako, Glostrup, Denmark) on consecutive sections [43].

Chromosomal number aberrations (CNAs) were assessed by metaphase CGH as described previously [36, 44]. In brief, crude tumor DNA from FFPE tissue was amplified by degenerate oligonucleotide primed-polymerase chain reaction (DOP-PCR) and labeled with another DOP-PCR using digoxigenin (DIG)-11-dUTP (Roche, Mannheim, Germany). The reference DNA was amplified from 50 ng of normal male or female DNA and labeled with biotin-dUTP (Roche). The probe mixture was denatured and hybridized to normal metaphase spreads (Vysis, Downers Grove, IL). Unhybridized probes were washed out, and the metaphase spread was incubated with a fluorescein isothiocyanate (FITC)-conjugated anti-DIG antibody (Roche) and rhodamine-conjugated avidin (Roche). Preparations were washed and counterstained with 4,6-diamino-2-phenylinodole (DAPI) in antifade solution. Red, green, and blue images were acquired, and ratios of fluorescence intensity along chromosomes were quantitated using the CytoVision® Analysis System (Applied Imaging, San Jose, CA).

*MGMT* promoter methylation was assessed by methylation-specific PCR (MSP) using the EZ DNA Methylation-Direct^TM^ Kit (Zymo Research Corp., Orange, CA) as described previously [45].

Mutation of the *IDH1/2* genes was assessed in three steps. First, formalin-fixed paraffin (FFP) sections were examined for IDH1R132H by immunohistochemistry with an anti-mutant IDH1 antibody (Dianova, Hamburg, Germany) [46]. For negative cases, exon 4 of the *IDH1* gene was amplified with previously described primers [47]. After purifying the PCR products, DNA sequencing was performed using the 3130xL Genetic Analyzer (Applied Biosystems, Foster City, CA, USA). Finally, in cases negative for an *IDH1* exon 4 mutation, exon 4 of *IDH2* was similarly amplified and sequenced [47].

Accumulation of p53 and loss of alpha-thalassemia/mental retardation syndrome X-linked (ATRX) expression were assessed by immunohistochemistry in “astrocytic” gliomas by original institutional diagnosis as molecular surrogates of astrocytic nature. FFP sections were examined for ATRX immunohistochemistry with anti-human ATRX (1:400, Sigma, HPA001906) and for p53 immunohistochemistry with anti-human p53 (1:50, Novocastra, DO-1). For the evaluation of p53, FFP sections from anaplastic astrocytoma resected in 1994 were used as positive control; staining of >5% nuclei for p53 was judged as positive. For the evaluation of ATRX, FFP sections from anaplastic oligoastrocytoma resected in 2012 were used as positive control, and staining of endothelial cells was considered as an internal positive control. Staining of >10% nuclei was judged as positive for ATRX [38].

### Statistical analysis

Patients were classified into two histopathological groups (oligodendroglial and astrocytic glioma). Demographic factors and the treatment and distribution of categorical factors such as CNAs were compared between groups by Fisher's exact test. The overall survival (OS) was calculated from the beginning of treatment, i.e., from the date of initial surgery for resection cases and from the first day of adjuvant therapy for biopsy cases. Survival curves were estimated and compared between groups using the Kaplan–Meier method and the log rank test. Significance levels for all tests were 2-sided and 0.05. All data were analyzed with IBM SPSS statistics 22.

## SUPPLEMENTARY MATERIAL TABLE


