# Supplementary material for: Molecular–genetic and clinical characteristics of gliomas with astrocytic appearance and total 1p19q loss in a single institutional consecutive cohort
Source: Oncotarget. 2015 May 11;6(18):15871–81. doi: 10.18632/oncotarget.3869 (PMC4599243; doi:10.18632/oncotarget.3869)
Supplement: Supplementary file 1 [file oncotarget-06-15871-s001.pdf]

Molecular−genetic and clinical characteristics of gliomas with astrocytic appearance and total 1p19q loss in a single institutional consecutive cohort

Supplementary Material

Table S1.Molecular and clinical characteristics of the 57 gliomas with total 1p19q loss.

Case	Age Sex	Resec-tion	Dx	CNAs	IDH	MGMT-MSP	P53	ATRX	OS	Alive Dead
1	26F	PR	OD	-1p/19q	1M	M	N/A	N/A	50≤	Alive
2	35F	STR	OD	-1p/19q, -4, -13q21-22	1M	U	N/A	N/A	187	Dead
3	47F	STR	OD	-1p/19q	1M	M	N/A	N/A	129≤	Alive
4	46M	STR	OD	-1p/19q	1M	M	N/A	N/A	33≤	Alive
5	23F	Biopsy	OD	-1p/19q, +19p	1M	U	N/A	N/A	76≤	Alive
6	53F	GTR	OD	-1p/19q, -14q22.3-ter, +17q22-ter	1M	N/A	N/A	N/A	87≤	Alive
7	40M	Biopsy	OD	-1p/19q, -18, +19p	1M	M	N/A	N/A	114≤	Alive
8	40M	GTR	OD	-1p/19q, -13q	1W	M	N/A	N/A	62≤	Alive
9	53M	Biopsy	OD	-1p/19q	1M	M	N/A	N/A	65≤	Alive
10	39F	STR	OD	-1p/19q, -4, +11	1M	M	N/A	N/A	87	Dead
11	56M	GTR	OD	-1p/19q, -1q24-31	1M	M	N/A	N/A	52≤	Alive
12	30M	STR	OD	-1p/19q	1M	U	N/A	N/A	64≤	Alive
13	63M	Biopsy	OD	-1p/19q	1M	M	N/A	N/A	36	Dead
14	37F	STR	OD	-1p/19q, +1q, -3, -6, +9, +11	1M	M	N/A	N/A	62≤	Alive
15	39M	STR	OD	-1p/19q	1M	M	N/A	N/A	171≤	Alive
16	53M	STR	OA	-1p/19q	1M	M	N/A	N/A	129≤	Alive
17	33F	PR	OA	-1p/19q	1M	U	N/A	N/A	73≤	Alive
18	34F	GTR	OA	-1p/19q, +7, +11, +17q,	1W 2M	M	N/A	N/A	60≤	Alive

				-18q						
19	32F	GTR	OA	-1p/19q, -4, -18q	1W 2M	M	N/A	N/A	105 ≤	Alive
20	27F	PR	OA	-1p/19q	1M	U	N/A	N/A	87≤	Alive
21	50M	Biopsy	OA	-1p/19q, -4, -9pter-21, -13q21-ter	1M	M	N/A	N/A	89≤	Alive
22	59F	GTR	OA	-1p/19q, -4q	1W 2M	M	N/A	N/A	100 ≤	Alive
23	31M	STR	OA	-1p/19q	1M	M	N/A	N/A	143 ≤	Alive
24	23M	PR	OA	-1p/19q, -4p15.3-15.1, -X	1M	N/A	N/A	N/A	151	Dead
25	36F	STR	AOD	-1p/19q, +11, -15q, -18q	1M	M	N/A	N/A	4≤	Alive
26	46F	PR	AOD	-1p/19q, -6pter-22, -13q21-31, -14q23-ter	1M	M	N/A	N/A	171 ≤	Alive
27	63M	Biopsy	AOD	-1p/19q, -4, -14q21-24	1M	M	N/A	N/A	49	Dead
28	64M	STR	AOD	-1p/19q, -4, +11, +17q, -18	1M	M	N/A	N/A	50≤	Alive
29	50M	Biopsy	AOD	-1p/19q, -4, +7, +8, +11	1M	U	N/A	N/A	238	Dead
30	36M	PR	AOD	-1p/19q, -4q28-31.3, +9q, +13q, +14qcen-23, -15q21.3-24.1, +15q26, -18	1M	M	N/A	N/A	293	Dead

31	52M	GTR	AOD	-1p/19q, -14q21-24	1M	M	N/A	N/A	51≤	Alive
32	22F	Biopsy	AOD	-1p/19q, -14q, +19p	1M	N/A	N/A	N/A	86	Dead
33	37F	STR	AOD	-1p/19q, -14q, +19p	1M	N/A	N/A	N/A	77	Dead
34	38M	STR	AOA	-1p/19q, -13q	1M	N/A	N/A	N/A	110≤	Alive
35	38M	STR	AOA	-1p/19q, -4p15.31-qter, +7q31-ter, +8q21.2-ter, -18q	N/A	M	N/A	N/A	90	Dead
36	41M	STR	AOA	-1p/19q, +17	1M	M	N/A	N/A	39≤	Alive
37	53M	Biopsy	AOA	-1p/19q, -14q, +22q	1W	U	N/A	N/A	86≤	Alive
38	40F	Biopsy	DA	-1p/19q	1M	M	Neg	Pos	20≤	Alive
39	37F	STR	DA	-1p/19q	1M	M	Neg	Pos	75≤	Alive
40	51M	PR	DA	-1p/19q	N/A	M	Neg	Pos	95	Dead
41	60M	Biopsy	DA	-1p/19q, -15q, -X	1M	M	Neg	Pos	143≤	Alive
42	52M	Biopsy	DA	-1p/19q, -14q21.3-ter	1M	N/A	Neg	Neg	213≤	Alive
43	62F	STR	DA	-1p/19q, -4, +7, +11q, -15q, -18q	1M	M	Neg	Pos	96≤	Alive
44	28F	GTR	DA	-1p/19q, -3p24-22, +7q	N/A	M	Neg	Pos	239≤	Alive
45	27M	PR	AA	-1p/19q, +11	1M	M	Neg	Pos	184	Dead
46	57M	Biopsy	AA	-1p/19q, -4	1M	M	N/A	N/A	103≤	Alive
47	63F	Biopsy	AA	-1p/19q, +1q, +4qcen-28, -4q31.2-t	1M	M	Neg	Pos	12	Dead

				er, -6q, +7, +9, -10, -13qcen-21, -14q, -17p12-cen						
48	52M	STR	AA	-1p/19q, +3q26.2-ter, -9pter-q33,+17, -18q21.2-22.1, +19p	1M	M	Neg	Neg	38	Dead
49	42M	PR	AA	-1p/19q, -6pter-21.32, +8, +9q34, +11q23-ter, +17q21.3-ter, +19p	1M	M	Neg	Pos	55≤	Alive
50	37M	STR	AA	-1p/19q, +7q, -9p21.3, +19p	N/A	M	Neg	Pos	94	Dead
51	44M	STR	AA	-1p/19q, +7, -10p	1M	M	Neg	Pos	176	Dead
52	27F	STR	AA	-1p/19q	1W 2M	M	Neg	Pos	23≤	Alive
53	28M	STR	AA	-1p/19q, +10, -18	1M	M	Neg	Neg	110 ≤	Alive
54	39M	STR	LGG	-1p/19q, -4	1M	M	N/A	N/A	162	Dead
55	31M	Biopsy	LGG	-1p/19q	1M	U	N/A	N/A	62≤	Alive
56	40M	Biopsy	LGG	-1p/19q, -19p	1M	M	N/A	N/A	61≤	Alive
57	54M	Biopsy	LGG	-1p/19q	1M	M	N/A	N/A	60≤	Alive

CNAs: choromosome copy number abberations, PR: partial removal, STR: subtotal removal, GTR: gross total removal, Dx: institutional diagnosis, OD: oligodendroglioma, AOD: anaplastic oligodendroglioma, OA: oligoastrocytoma, DA: diffuse astrocytoma, AA: anaplastic astrocytoma, LGG: unclassified low grade glioma, IDH M: mutated, IDH W: wild-type, MGMT-MSP: methylation-specific PCR of the *MGMT* gene, MSP M: methyled, MSP U: unmethyled, Pos: positive, Neg: negative, OS: overall survival (months)
